# Responsive Liquid Metal Droplets: From Bulk to Nano

**DOI:** 10.3390/nano12081289

**Published:** 2022-04-10

**Authors:** Minghui Duan, Xiyu Zhu, Xiaohui Shan, Hongzhang Wang, Sen Chen, Jing Liu

**Affiliations:** 1 Department of Biomedical Engineering, School of Medicine, Tsinghua University, Beijing 100084, China; dmh17@mails.tsinghua.edu.cn (M.D.); zxy19@mails.tsinghua.edu.cn (X.Z.); sxh19@mails.tsinghua.edu.cn (X.S.); wanghz15@tsinghua.org.cn (H.W.); 2 Beijing Key Laboratory of Cryo-Biomedical Engineering, Technical Institute of Physics and Chemistry, Chinese Academy of Sciences, Beijing 100190, China

**Keywords:** liquid metal, droplets, stimuli-responsive materials, smart matter, motors

## Abstract

Droplets exist widely in nature and play an extremely important role in a broad variety of industrial processes. Typical droplets, including water and oil droplets, have received extensive attention and research, however their single properties still cannot meet diverse needs. Fortunately, liquid metal droplets emerging in recent years possess outstanding properties, including large surface tension, excellent electrical and thermal conductivity, convenient chemical processing, easy transition between liquid and solid phase state, and large-scale deformability, etc. More interestingly, liquid metal droplets with unique features can respond to external factors, including the electronic field, magnetic field, acoustic field, chemical field, temperature, and light, exhibiting extraordinary intelligent response characteristics. Their development over the past decade has brought substantial breakthroughs and progress. To better promote the advancement of this field, the present article is devoted to systematically summarizing and analyzing the recent fundamental progress of responsive liquid metal droplets, not only involving droplet characteristics and preparation methods, but also focusing on their diverse response behaviors and mechanisms. On this basis, the challenges and prospects related to the following development of liquid metal droplets are also proposed. In the future, responsive liquid metal droplets with a rapid development trend are expected to play a key role in soft robots, biomedicine, smart matter, and a variety of other fields.

## 1. Introduction

In the morning, the blades of roadside grass are covered in glittering water droplets, which is familiar to everyone. In fact, droplets not only exist widely in nature, but also play an increasingly indispensable role in production and life. Here, droplets can be considered as small liquid particles that are dispersed in immiscible liquid or gas by stirring or jetting, and so on. To minimize the energy of the system, droplets will merge when the continuous phase film coated on the surface of the droplets breaks by colliding or extruding. Previous research revealed that droplets can remain stable for a longer time with the addition of surfactant [1,2], which is beneficial for practical applications. Traditionally, droplets can be fabricated by nozzles, small holes, and agitators in industrial production. In contrast to continuous liquid, dispersed droplets have larger surface areas under the same volume, and thus droplets can accelerate the speed of both physical and chemical reactions at the interface, including heat exchange [3,4,5], extraction, and redox reaction [6,7]. For example, in a diesel engine, diesel oil is atomized into droplets through the nozzle and fully mixed with the air to ensure full combustion [8]. Currently, droplets are widely used in microfluidics, motors, atomization, cell biology, etc. [9,10,11]. 

**Figure 1:**
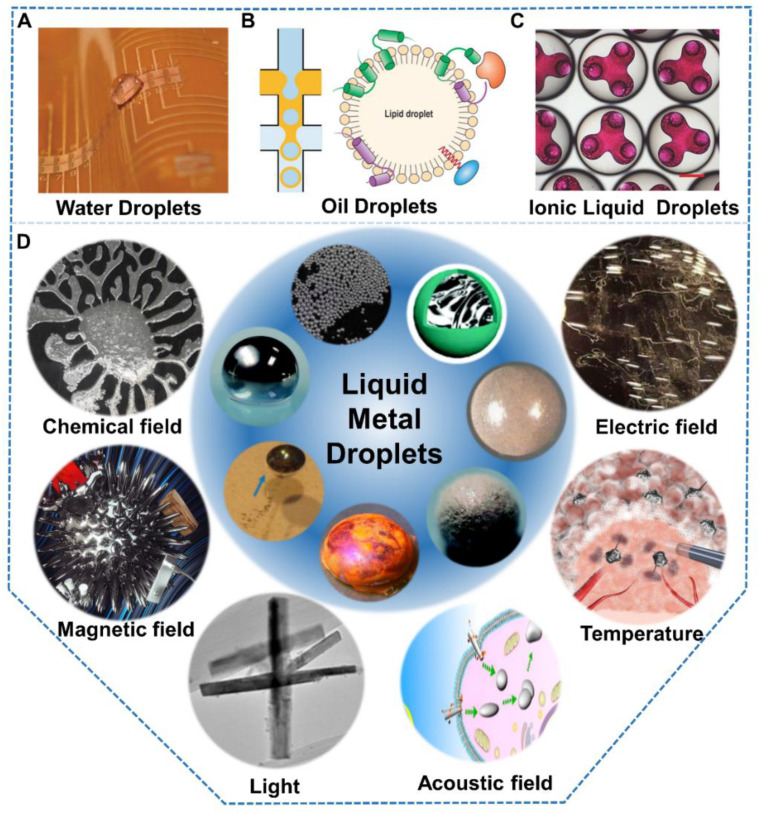
Different droplets. Common droplets including (**A**) water droplets. Reprinted with permission from ref. [22]. Copyright 2009, John Wiley and Sons. (**B**) oil droplets. Reprinted with permission from ref. [16]. Copyright 2018, John Wiley and Sons. Reprinted with permission from ref. [23]. Copyright 2021, John Wiley and Sons. (**C**) ionic liquid droplets. Reprinted with permission from ref. [19]. Copyright 2021, John Wiley and Sons. (**D**) Liquid metal droplets from structure to responses induced by different simulations. Reprinted with permission from ref. [24]. Copyright 2019, Spring Nature. Reprinted with permission from ref. [25]. Copyright 2018, John Wiley and Sons. Reprinted with permission from ref. [26]. Copyright 2017, The Royal Society of Chemistry. Reprinted with permission from ref. [27]. Copyright 2018, American Chemical Society. Reprinted with permission from ref. [28]. Copyright, 2015, Elsevier. Reprinted with permission from ref. [29]. Copyright 2018, Elsevier. Reprinted with permission from ref. [30]. Copyright, 2015, The Royal Society (U.K.) Reprinted with permission from [31]. Copyright 2017, American Chemical Society. Reprinted with permission from ref. [32]. Copyright, 2020, John Wiley and Sons. Reprinted with permission from [33]. Copyright 2018, American Chemical Society.

Typical droplets include water droplets (Figure 1A), oil droplets (Figure 1B), and ionic liquid droplets (Figure 1C). Water droplets are most common in everyday life, such as in raindrops, spraying pesticides, steam after water boiling, and so on. Meanwhile, water droplets have been widely used in multiple fields, such as harvesting energy from droplets [12,13,14]. Oil is a general term for a glass of hydrophobic liquid. In addition to its application in internal combustion engines, oil combined with water can be used in microfluidics [9,15]. Lipid droplets are also a kind of oil droplet, which play an essential role in cell composition and function [11,16]. Ionic liquid usually refers to room temperature ionic liquid, which is entirely composed of cations and anions. Due to the characteristics of low vapor pressure, flame retardation, and easy recovery, ionic liquids are widely used as substitutes for organic solvents [17,18]. Furthermore, ionic liquids can be made into droplet arrays to achieve low consumption and efficient chemical reactions [17,19]. Droplets in the microchannel are used to isolate reactants to achieve low-cost and high-throughput analysis [20,21]. Although common droplets are widely used, their performance is still relatively single, and they are still insufficient in meeting emerging needs, such as manufacturing of multi-functional machines. 

Significantly different from traditional liquid, as illustrated in Table 1, room temperature Ga-based liquid metal (LM) combining the characteristics of fluid and metal has recently attracted increased attention [34,35,36,37,38]. They possess excellent fluidity [39,40], extraordinary electrical conductivity [41,42,43], high thermal conductivity [44,45,46], and low toxicity [47,48,49], and therefore show great potential in soft and flexible electronics [50,51,52], biomedicine [53,54,55], thermal interface materials [56,57,58], chemical catalysis [59,60,61,62], and soft robots [63,64,65]. As a metal fluid, LM is immiscible with water, oil, and ionic liquids, which means that bulk LM could be divided into small droplets in other immiscible solutions [2]. Furthermore, due to its relatively low melting point and extremely high boiling point, LM has the widest liquid temperature range compared to other common droplets, which is beneficial to application. Moreover, in contrast to common droplets, LM droplets possess the highest surface tension of over 500 mN/m, so they are easier to maintain spherical on the substrate [66]. Therefore, one can conclude that LM droplets with spherical shape in a wide temperature range can be obtained. Compared with bulk LM, LM droplets coupled with distinctive characteristics exhibit rich response behaviors [67], which are of great significance for expanding their application range. Actually, it is equally important that LM droplets have a good electric conductivity and thermal conductivity, which is exactly the basis of multi-responses materials. It is a very unusual discovery that self-driven LM droplets, which used to be science fiction, have now become a reality through feeding with aluminum (Al) [68]. Furthermore, LM droplets even without fuel will transform or move under various stimulation, including chemical factors, electric field, magnetic field, temperature, light, and ultrasound [31,34,69,70,71,72], as illustrated in Figure 1D. These excellent physical properties and multi-response characteristics determine LM droplets to have great potential in intelligent machine and drug delivery.

With extensive attention paid to responsive LM droplets, massive achievements have emerged. To better promote the following advancement, this review is dedicated to systematically summarizing and analyzing the recent progress of responsive LM droplets. Firstly, we will introduce the fabrication methods of LM droplets. Then, the characteristics of LM droplets fabricated through different strategies will be introduced, respectively. Next, the classification based on structure of LM droplets will be proposed. Furthermore, the responses induced by different stimulations will be highlighted. Finally, the challenges and the outlook will be discussed. 

## 2. Liquid Metal Droplets

### 2.1. Fabrication of Pure Liquid Metal Droplets

**Figure 2:**
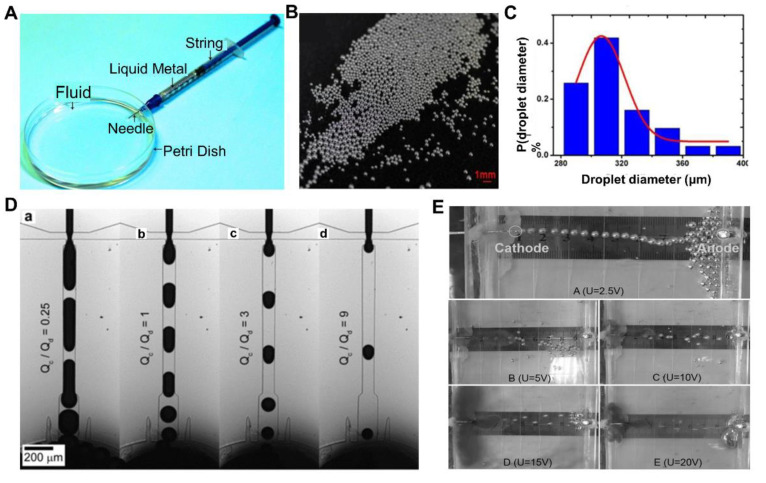
Fabrication methods of liquid metal (LM) droplets. (**A**) Device of fluidic jetting for LM droplet fabrication. Reprinted with permission from ref. [2]. Copyright 2013, John Wiley and Sons. (**B**) Optical image of LM droplets. Reprinted with permission from ref. [2]. Copyright 2013, John Wiley and Sons. (**C**) Histogram of droplet diameter distribution fabricated by fluidic jetting. Reprinted with permission from ref. [2]. Copyright 2013, John Wiley and Sons. (**D**) Micrographs of LM droplet fabrication at different flow rate ratios. Here, Q_c_ is the volumetric flow rate of the continuous phase, Q_d_ is the volumetric flow rate of LM. Reprinted with permission from ref. [78]. Copyright 2012, John Wiley and Sons. (**E**) The electro-hydrodynamic shooting phenomenon at different voltages. Reprinted with permission from ref. [83]. Copyright 2014, AIP Publishing.

LM droplets can be obtained in both micro and nanoscale, due to the fast development of nanoscience and fabrication techniques. In general, pure LM droplets can be fabricated by fluidic jetting, or microfluidic flow focusing. The method chosen for fabricating can be decided quickly by the size of target droplets. Fluidic jetting is a convenient and fast method for obtaining LM droplets. Yu et al. [2] proposed a method of injecting LM into water solution with added surfactant by using a syringe at room temperature (Figure 2A). The mechanism, which was revealed in this research, is that the surface tension of jetting LM stream overcomes the viscous shear stress, causing the bulk LM to break into micro droplets. As illustrated in Figure 2B, the LM droplets fabricated by this method were stable and can be obtained quickly. The key to prevent the LM droplets from coalescing and merging together lies in the introduction of the surfactant sodium dodecyl sulfate (SDS) [2]. The jetting flow velocity of LM and the diameter of the syringe needle are two factors which can influence the size of the droplets, and the droplets are mostly at the micrometer scale (Figure 2C). According to specific need, such a method was also demonstrated to work well to produce wires or a porous structure. In the following years, more fabrication advancements were also reported [73]. Overall, fluidic jetting is straightforward, low cost, and has an important value in LM droplet fabrication [28,74]. However, the droplet size is difficult to reduce due to the limitation of the needle size.

Microfluidics are widely used in many application scenarios, such as biological analysis, chemical synthesis, single-cell analysis, and tissue engineering [75,76]. However, the fluids mainly used in microfluidics are water and oil [77]. LM micro droplets can be fabricated by microfluidic flow focusing as well, because of the satisfactory liquidity of LM at room temperature [78,79]. A microfluidic chip was presented, which integrates continuous generation of micro scale LM droplets in glycerol [80]. Galinstan micro droplets can be produced continuously in glycerol. The NaOH solution is to remove the oxide layer of LM droplets and prevent further oxidation [81]. Moreover, the HCl solution has a similar function and can be used in microfluidics as well [82]. The main factors affecting droplet size are the channel dimensions and flow rates of fluids. As illustrated in Figure 2D, Hutter et al. [78] revealed that different flow rate ratios of EGaIn and the continuous phase had an influence on the droplet diameter. Water containing 20 wt% polyethylene glycol (PEG) and 5 wt% sodium dodecyl sulfate (SDS) was used as the continuous phase and the diameter of the nozzle was 40 μm. With the increase of Q_c_/Q_d_ value, where Q_c_ is the volumetric flow rate of the continuous phase and Q_d_ is the volumetric flow rate of LM, the LM droplet volumes decreased quickly and the droplet diameters decreased as well. The diameters of LM droplets fabricated by microfluidic flow focusing can be decreased to <30 μm by tuning the size of microchannel. Compared to fluidic jetting, microfluidic flow focusing is a more stable method that can fabricate LM droplets continuously, where the size of droplets is more controllable as well.

Fang et al. [83] reported that bulk LM can be separated into discrete droplets by electro-hydrodynamic shooting. When the capillary tube filled with bulk LM was on a direct current (DC) electric field, the bulk LM shot into the solution and formed droplets continuously, as illustrated in Figure 2E. The shooting velocity increased as the voltage increased, and the droplet size depended on the diameter of the capillary nozzle. Electro-hydrodynamic shooting provides a convenient way to produce LM droplets quickly, but the diameter of the capillary tube limits the size of droplets.

**Figure 3:**
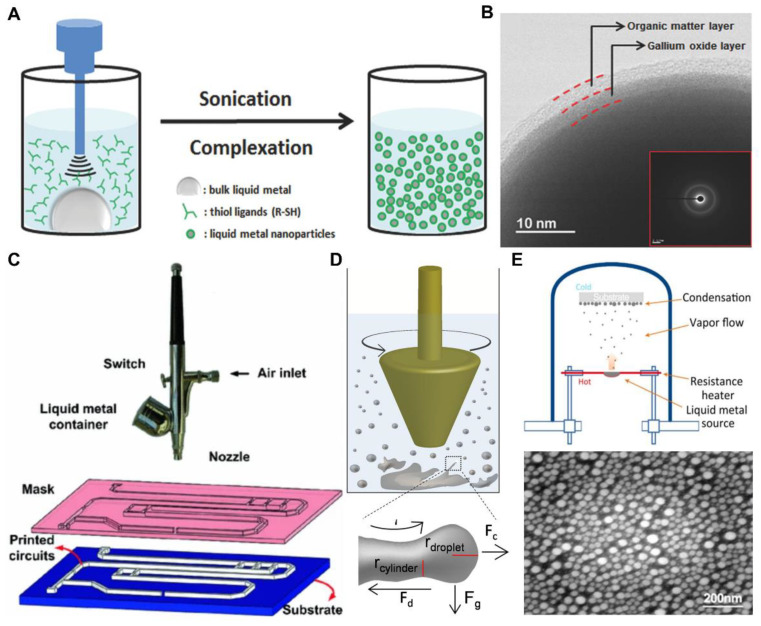
Fabrication methods of liquid metal (LM) droplets. (**A**) Schematic illustration of LM nanodroplets fabrication by ultrasonic cavitation. Reprinted with permission from ref. [87]. Copyright 2016, John Wiley and Sons. (**B**) The core-shell structure of LM nanodroplets fabricated by ultrasonic cavitation. Reprinted with permission from ref. [87]. Copyright 2016, John Wiley and Sons. (**C**) Schematic diagram of atomized spraying equipment for fabrication of LM droplets. Reprinted with permission from ref. [90]. Copyright 2013, Spring Nature. (**D**) Schematic diagram of SLICE and the structure of droplets. Reprinted with permission from ref. [91]. Copyright 2014, American Chemical Society. (**E**) Preparing LM droplets through physical vapor deposition technique. Reprinted with permission from ref. [92]. Copyright 2018, Elsevier.

Metal droplets can be fabricated by ultrasonic cavitation when low-melting-point metals mix with hot silicone [84,85]. Similarly, ultrasonic cavitation is also suitable for fabrication of LM micro/nanodroplets [86,87,88,89]. Organic solvent added to bulk LM forms an immiscible liquid system, and the ultrasonication process induces the dispersion of LM and formation of droplets. As illustrated in Figure 3A, Ren et al. [87] reported stable Galinstan droplets with average diameters of 110 nm, which can be suspended in solution for several weeks. The ultrasonication process induced the rapid oxidation of Ga, and the oxide layer covered the droplet surfaces. Moreover, the thiol ligands (R-SH) added into the ethanol solution formed an organic matter layer on the Ga oxide layer by a self-assembly process (Figure 3B). The double layer structure protected the LM nanodroplets from coalescence in the neutral solution or atmosphere. The LM droplet diameters depend on the temperature and ultrasonication time [88]. A smaller LM droplet can be fabricated by lower temperature or longer ultrasonication time. However, there is a size limit for LM droplets under different ultrasonication time. Ultrasonic cavitation is a widely used method for fabrication of LM nanodroplets, and is worth further research in addressing how to break their size limit.

Zhang et al. [90] reported a convenient method for fabricating LM droplets by utilizing an airbrush (Figure 3C). The bulk LM was rapidly squeezed through the fluid nozzle by high-pressure air, and then separated into LM droplets. Depending on the diameter of the nozzle, the droplet diameter ranged from 700 nm to 50 μm. The mechanisms of atomized spraying and fluidic jetting are similar; the bulk LM separates when squeezed out of a narrow nozzle and forms micro/nanodroplets due to its high surface tension. Atomized spraying provides a convenient and fast way of LM droplet fabrication in the atmosphere at room temperature. However, because the droplets coalesce rapidly and are difficult to collect, atomized spraying is commonly used to fabricate LM electronics on flexible substrate. Shearing liquids into complex particles (SLICE) is a simple method used to make LM droplets by utilizing emulsion shearing with oxidation. As illustrated in Figure 3D, by utilizing mechanical force, bulk LM was broken to small droplets with concomitant surface oxidation and functionalization [91,93]. The LM droplets fabricated by SLICE can be 6.4 nm to over 10 μm in diameter, which is related to the magnitude of shear force. SLICE provides a low-cost, green, facile, and versatile method that can obtain LM micro/nanodroplets of tunable sizes, shapes, compositions, and surface morphologies. However, SLICE is worth being further investigated because the distribution of droplet diameter is uneven. Furthermore, physical vapor deposition based on evaporation and deposition is also an effective preparation method of LM nano droplets, and the droplet size of LM droplets is small and uniform. As shown in Figure 3E, Yu et al. [92] report a way to synthesize surfactant-free LM nanodroplets with controlled particle size on a variety of substrates through a facile physical vapor deposition method. 

### 2.2. Liquid Metal Droplets with Core-Shell Structure

**Figure 4:**
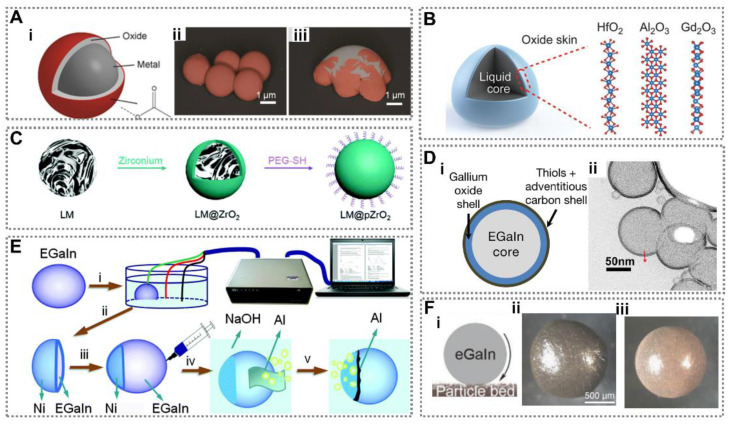
Liquid metal (LM) droplets with core-shell structure. (**A**) Schematic of LM droplets with core-shell structure and the false-colorized images of droplets to highlight the fractured oxide layer. Reprinted from Ref. [93]. (**B**) A cross-sectional diagram of a LM droplet, with possible crystal structures of different oxide layers. Reprinted with permission from ref. [96]. Copyright 2017, The American Association for the Advancement of Science (**C**) Schematic diagram of the synthesis route of LM nanodroplets. Reprinted with permission from ref. [24]. Copyright 2019, Spring Nature. (**D**) Schematic diagram and pretreated scanning transmission electron microscopy (STEM) image of LM droplets with core-shell structure. Reprinted with permission from ref. [97]. Copyright 2017, American Chemical Society. (**E**) Schematic diagram of fabrication of the magnetic soft motor with core-shell structure [98]. Copyright 2016, The Royal Society of Chemistry (**F**) Formation of thin porous particle networks at LM droplet interface. Reprinted with permission from ref. [25]. Copyright 2018, John Wiley and Sons.

LM droplets with core-shell structure are a nanoscale ordered assembly structure obtained by LM core coated by another nanomaterial through chemical bonds or other forces [39,94]. By utilizing surface modification, LM droplets with core-shell structure can be obtained on the basis of the pure LM droplets. The properties of core-shell structure depend on the core and shell materials, concentrate on the advantages, and make up for disadvantages as well. Moreover, because of forming rapidly, the native oxides of Ga play an important role between LM core and shell material. Generally, Ga and its alloy are covered by a thin oxide layer as Ga can be oxidized easily and rapidly when exposed to the air or neutral solution [95]. Contrarily to the strong surface tension of LM, the oxides of Ga have a much smaller surface tension and greatly change the wettability between LM and substrate. For LM droplets with core-shell structure, the oxide layer can also be considered like a shell, which can connect other materials stably. As illustrated in Figure 4A, the LM droplet with core-shell structure fabricated by SLICE is composed of LM core, oxide layer and surfactant shell [93]. The oxide layer highlighted by false-colorization is fractured by an external force. 

Zavabeti et al. [96] created a variety of low-dimensional metal oxides by utilizing LM droplets with core-shell structure as a reaction environment. As illustrated in Figure 4B, 1 wt% of hafnium (Hf), aluminum (Al), or gadolinium (Gd) was alloyed into LM droplets, and their oxides were easier to form than oxides of Ga. By touching the LM droplets with a solid substrate, the metal oxides adhered to the substrate and were separated from droplets because of the different phase between the LM droplets and solid oxides. Moreover, LM droplets with core-shell structure also can be used as nanomedicines by modifying their surfaces with functional layers. ZrO_2_ coated LM nanodroplets were fabricated for photothermal therapy (Figure 4C) [24]. The ZrO_2_ shell can effectively prevent the droplets from coalescence and size variation, and PEG was used to improve the biocompatibility of the droplets. Thus, droplets warm up rapidly under near-infrared (NIR) laser radiation. LM droplets with core-shell structure are confirmed to be a promising vehicle for nanotheranostics, and have attracted widespread attention. In addition, the oxide layer of LM droplets has been measured from 0.5 nm to 5 nm in thickness [99]. As the LM droplet diameter decreases, the mass fraction of LM core decreases as well. It is possible that Ga is completely oxidized and that LM droplets lose original properties and functions. Farrel et al. reported a method to successfully control the growth of a Ga oxide layer on LM nanodroplets by adding thiolated molecules (Figure 4D) [97]. Their research indicated thiolated molecules can moderate the growth of Ga oxide via competition with oxygen for surface sites. The results are of great significance for fabricating multifunctional nanodroplets.

LM droplets with core-shell structure also can be fabricated by electroplating. Zhang et al. reported a magnetic soft motor with core-shell structure by electroplating a Ni cap [98]. As illustrated in Figure 4E, Al foil was added to the droplets as the on-board fuel. The LM droplet can continuously move with a velocity of 3 cm·s^−1^ for hours without an external energy source. As a self-propelled droplet motor, it can be controlled under an applied magnetic field or electric field. However, the accurate control of this droplet movement needs to be further studied and has important significance in drug delivery. Tang et al. synthesized a LM droplet with a LM core and a Cu shell by electrochemical reduction [25]. The preoxidized Cu nanoparticles were first coated on a LM droplet to obtain a LM marble. After alkaline solution was added to the system, the color and shape of the LM marble changed, which is a signature phenomenon of the electrochemical welding process (Figure 4F). This research demonstrated an effective method to cross-link various types of particles by the redox reaction. Thus, researches indicate that LM droplets with core-shell structure can concentrate the properties of core and shell materials. Multifunctional LM droplets can be obtained by adjusting the properties of either core or shell material. In the future, it is necessary to further study the core-shell interface to obtain more stable droplets. Moreover, it is also expected to achieve more functions by manufacturing droplets with more complex structures, such as controllable motors, droplet micro reaction environments, nanorobots, and so on.

### 2.3. Liquid Metal Marbles

**Figure 5:**
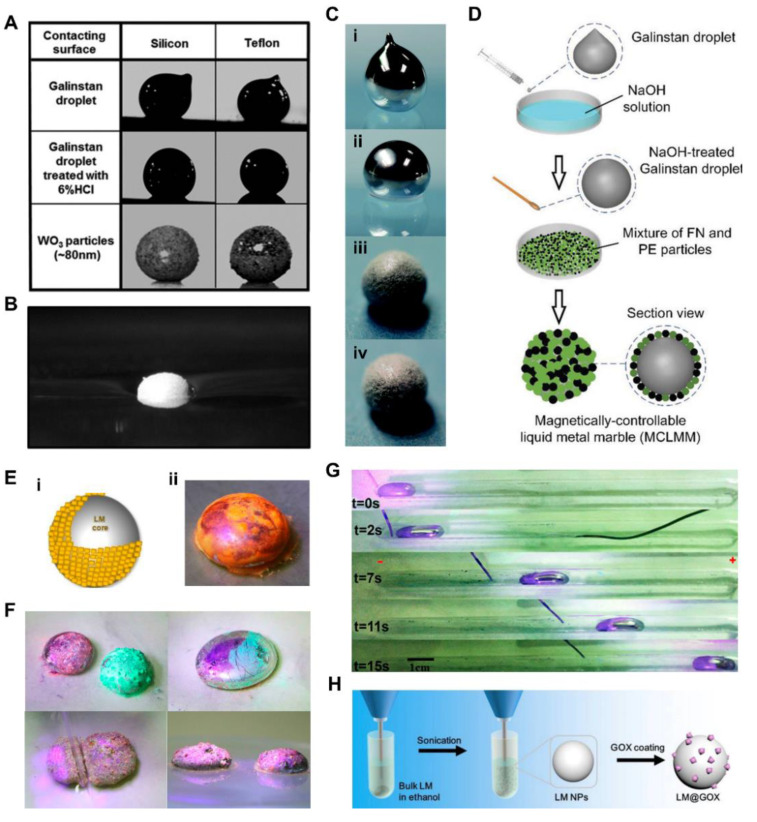
Liquid metal (LM) marbles. (**A**) Images of a LM droplet with naturally formed native oxide layer in ambient air, LM droplets treated with diluted HCl solution and WO_3_ powder coated LM on different surfaces. Reprinted with permission from ref. [102]. Copyright 2012, John Wiley and Sons. (**B**) Sigmacote treated Al_2_O_3_ powder (≈9 μm) coated LM droplet floating on water. Reprinted with permission from ref. [102]. Copyright 2012, John Wiley and Sons. (**C**) Optical images of (i) pure LM droplet, (ii) LM droplet treated with NaOH solution, (iii) LM droplet stabilized by PTFE particles, (iv) LM droplet treated with NaOH solution and stabilized by PTFE particles. Reprinted with permission from ref. [26]. Copyright 2017, The Royal Society of Chemistry. (**D**) Schematic diagram of the fabrication of the magnetically controllable LM marble. Reprinted with permission from ref. [103]. Copyright 2019, John Wiley and Sons. (**E**) Schematic diagram and optical image of the LM fluorescent marble. Reprinted with permission from ref. [27]. Copyright 2018, American Chemical Society. (**F**) The coalescence and separation of LM marbles. Reprinted with permission from ref. [27]. Copyright 2018, American Chemical Society. (**G**) The movement of the fluorescent LM marbles in a basic electrolyte (U = 15 V). Reprinted with permission from ref. [27]. Copyright 2018, American Chemical Society. (**H**) The preparation process of LM marbles coated with GOX. Reprinted with permission from ref. [104]. Copyright 2019, Elsevier.

LM marbles are droplets that micro- or nanoparticles coat on the LM core. Generally, LM marbles belong to LM droplets with core-shell structure and are a rather prominent category, so we introduce them in a separate section. The oxide layer of LM formed easily in ambient air has a much lower surface tension than LM, and can adhere strongly to glass, cloth, and silicone [100]. In addition, LM is highly corrosive to other metals, which limits its practical application [101]. To deal with these problems, researchers coated LM droplets with various micro- or nanopowders. Sivan et al. [102] introduced LM marbles formed by coating nanoscale powders on the surface of LM droplets. By rolling the LM droplets on a powder bed, LM marbles coated with insulators and semiconductors such as SiO_2_ and WO_3_ can be obtained. As illustrated in Figure 5A, LM droplets formed tips because of the surface oxidation of LM droplets. However, LM marbles coated with WO_3_ powder maintained their original spherical shapes, similarly to those treated with HCl solution. In addition, LM marbles coated with Al_2_O_3_ powder can suspend on the surface of water (Figure 5B). 

As illustrated in Figure 5C, Chen et al. [26] reported a highly elastic and movable LM marble by coating LM core with polytetrafluoroethylene (PTFE) particles. The results of bouncing tests indicated such LM marbles can bounce nine times after falling from the initial height. However, the LM droplet falling from the same initial height stuck to the substrate without any bouncing. Furthermore, the LM marble could scroll on a substrate with an inclined angle of 14°. Chen et al. [103] presented a magnetically controllable LM marble, which was coated with ferronickel (FN) and polyethylene (PE) microparticles. The LM core was treated with NaOH solution to remove its oxidized layer and then mixed with FN and PE particles (Figure 5D). The research indicated the LM marbles have no corrosive residues on the surfaces of different materials after 12 h. In particular, such LM marbles can be controlled by a magnetic field due to the addition of the FN particle.

Further, Liang et al. [27] reported a fluorescent LM marble as a transformable biomimetic chameleon. As illustrated in Figure 5E, fluorescent LM marbles can be fabricated by encasing them with fluorescent nanoparticles. The exhibited color of such LM marble depended on the fluorescent particles. The different colored fluorescent LM marbles could merge together to form a biggish ultimate multicolor LM marble (Figure 5F). One entire LM marble could be divided into several small LM marbles as well. The fluorescent particles could also be released from LM marbles under an electric field by adjusting the distribution of the oxide layer. Furthermore, the electric field could lead to a controllable movement of the fluorescent LM marble in a basic electrolyte as well (Figure 5G). LM marbles can also be used as nanomedicine [39,53]. Hu et al. [104] synthesized a LM marble coated with glucose oxidase (GOX) for tumor treatment (Figure 5H). GOX can consume intratumoral glucose by oxidation in order to suppress tumor growth. LM has an outstanding photothermal conversion ability and good biocompatibility. By utilizing such LM marbles, Hu et al. brought the two methods together and found that combinational therapy including starvation and photothermal therapy realized outstanding therapeutic effects. Generally, LM marbles can greatly improve the mechanical properties of LM droplets and avoid the corrosivity of LM. In the future, it is necessary to research the method of further kinds of nanoparticles that can be coated on the surface of LM droplets.

### 2.4. Self-Powered Liquid Metal Droplet Motors

**Figure 6:**
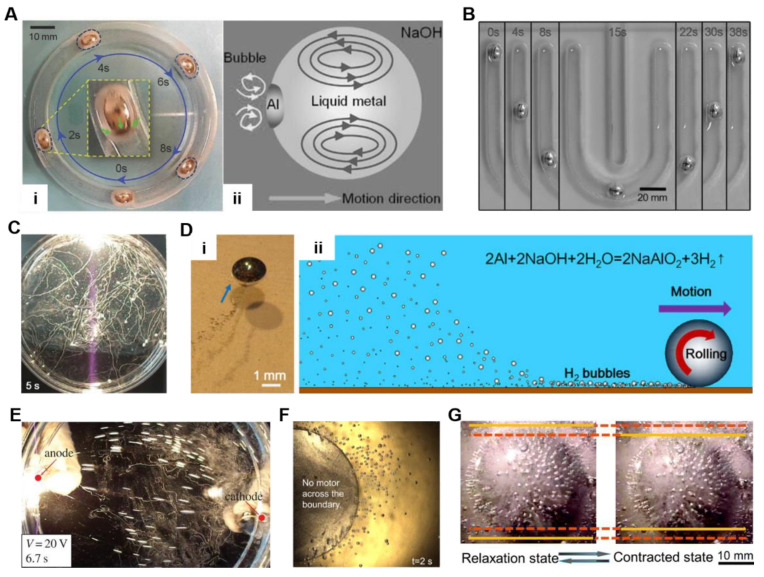
Self-powered liquid metal (LM) droplets. (**A**) Self-fueled LM droplet running in a circular channel and the schematic diagram of the propulsion mechanism. Reprinted with permission from ref. [68]. Copyright, 2015, John Wiley and Sons. (**B**) Self-driven motion of LM in U-shaped channel. Reprinted with permission from ref. [68]. Copyright, 2015, John Wiley and Sons. (**C**) Irregular motion of LM droplets. The curves in the figure are the trajectory of droplets. Reprinted with permission from ref. [28]. Copyright, 2015, Elsevier. (**D**) Optical image and schematic diagram of self-powered LM droplet motor. Reprinted with permission from ref. [28]. Copyright, 2015, Elsevier. (**E**) The controllable motion of LM droplet motors. Reprinted with permission from ref. [30]. Copyright, 2015, The Royal Society (U.K.). (**F**) LM droplet motors trapped in the boundary zone of the magnet. Reprinted with permission from ref. [106]. Copyright, 2015, AIP publishing. (**G**) The oscillation process of LM droplets in one oscillation cycle. Reprinted with permission from ref. [107]. Copyright, 2019, The Royal Society of Chemistry.

A self-propulsion phenomenon of LM droplets has been discovered by adding Al. Zhang et al. [68] introduced a self-fueled LM motor as a biomimetic mollusk. The LM motor could “eat” Al food and move spontaneously in NaOH solution for more than 1 h (Figure 6A,B). The bubble recoil force and the surface tension gradient induced by the galvanic reaction propelled the motor forward. The movement direction is opposite to the bubble injection direction, and the velocity can reach up to 5 cm·s^−1^. A large LM motor can be divided into several small ones that can maintain original motion characteristics. In contrast, many small LM motors will coalesce if they collide in the movement [74,105]. Yuan et al. [28] found the random motion of LM motors resembled the Brownian motion (Figure 6C). Millimeter scale motors were fabricated by injecting the LM composed of 1 wt% Al and 99 wt% GaIn_10_ into NaOH solution. As illustrated in Figure 6D, the LM motors could roll forward on the substrate propelled by the bubble recoil force.

Under an electric field, these LM droplet motors could move in a controllable direction and speed (Figure 6E) [30]. The motion trajectory was similar to the distribution of the electric field in solution, and the velocity could reach up to 43 cm·s^−1^ under 20 V in a channel filled with aqueous solution. It is an effective method to drive the droplet motor to move at high speed. If the electric field is changed into a magnetic field, the self-powered LM droplet motors will become trapped in the boundary zone of the magnet due to the Lorentz force [106] (Figure 6F). Furthermore, the LM droplets with added Al could realize high frequency self-powered oscillations via redox reaction when placed on an iron plate (Figure 6G) [107]. The oscillation behavior had a frequency of 8 Hz and shows the potential for developing self-powered soft oscillation. Generally, the addition of Al provides a novel strategy for fabricating self-powered LM droplet machines. This method is also expected to promote the development of multifunctional soft droplet machines. 

## 3. Response Behaviors of Liquid Metal Droplets

Based on the excellent characteristics of LM, after recent development, the rich response behaviors of LM droplets have been found and explained. So far, multiple factors have been demonstrated to induce LM to achieve specific responsive behaviors, including the chemical field, electronic field, magnetic field, acoustic field, temperature, and light. The mechanism lying behind the response behaviors has also been revealed. Different external stimuli usually show their own characteristics. Based on these response behaviors, liquid metal droplets have also been applied in many fields. The summary of the response characteristics of these external stimuli can be seen in Table 2, which will be more specifically introduced later.

### 3.1. Chemically Induced Response

**Figure 7:**
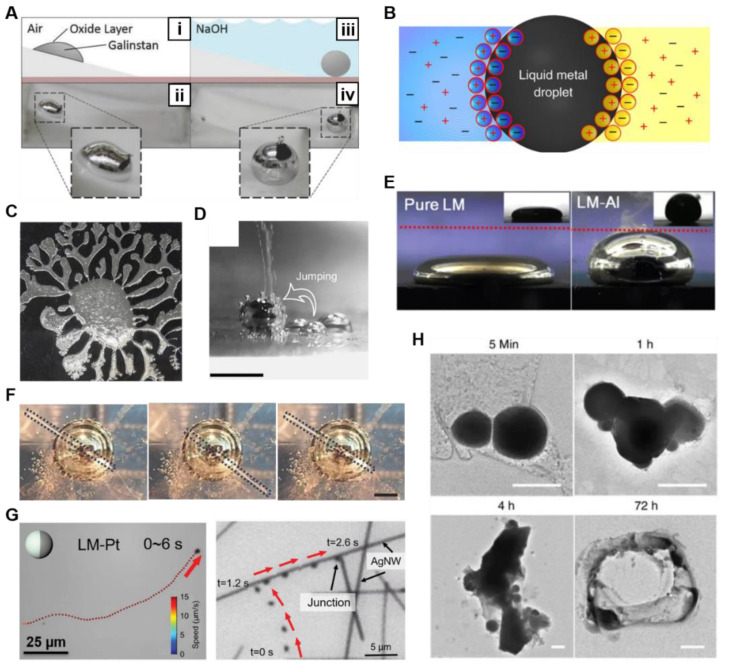
Chemically induced responses. (**A**) The different morphology of liquid metal (LM) droplet in air and NaOH solution, respectively. Reprinted with permission from ref. [114]. Copyright 2017, John Wiley and Sons. (**B**) Schematic diagram of the LM droplet and the EDL forming on the surface of the LM droplet. Reprinted from Ref. [115]. (**C**) The image of LM fractal phenomenon induced by synergistic oxidation. Reprinted with permission from ref. [29]. Copyright 2018, Elsevier. (**D**) The side view of LM droplet jumping. The scale bar is 500 μm. Reprinted with permission from ref. [117]. Copyright 2016, AIP Publishing. (**E**) The addition of Al increased surface tension of LM droplet on graphite substrate. Reprinted from Ref. [121]. (**F**) Top view of oscillation behavior of copper wire in a LM droplet. Reprinted from Ref. [122]. (**G**) Microwelding of silver nanowires by self-propelled LM droplets. Reprinted with permission from ref. [123]. Copyright 2022, John Wiley and Sons. (**H**) The fusion and degradation process of LM nanodroplets triggered by acid. The scale bars are 100 nm. Reprinted from Ref. [124].

LM droplets can adhere to various substrates due to the formation of oxide layers coated on the droplets. However, the oxide layer can be removed by HCl and NaOH solution [26,102]. After the removal of the oxide layer, the LM droplet will lose its adhesion to the substrate and return to a sphere under its strong surface tension (Figure 7A) [114]. In contrast, NaOH solution affords faster oxide removal than HCl [66]. Furthermore, the ionic balance at the interface between LM and solution can be reached by forming an electrical double layer (EDL). Zavabeti et al. [115] introduced the self-propulsion of LM induced by ionic imbalance. By changing the electrolyte surrounding the LM droplet, the EDL on the surface of the droplet changed and directly led to the difference of surface tension, which can drive the droplet to move spontaneously (Figure 7B). In addition, the difference in surface tension can be caused by the uneven oxidation on the surface of the droplets. Gough et al. presented a method to drive the LM droplets via redox reaction [116]. The oxides formed on the side in contact with the solution via redox reaction, and then the LM could flow into a channel due to the surface tension difference. Furthermore, Chen et al. presented a LM fractal phenomenon induced by synergistic oxidation (Figure 7C) [29]. The LM droplet placed on the graphite in the NaOH solution formed a galvanic cell, in which Ga lost electrons and was oxidized.

Meanwhile, the addition of hydrogen peroxide (H_2_O_2_) led to further synergistic oxidation. The surface tension difference induced by synergistic oxidation led to the intriguing LM fractal phenomenon. The mechanism of above large-scale transformation and actuation is the Marangoni effect induced by the uneven oxidation or EDL distribution. In addition, by adding nickel (Ni) particles, the LM droplet jumped in the NaOH solution due to the generation and accumulation of hydrogen (Figure 7D) [117]. The LM droplet can also realize a self-oscillating system when placed in the NaOH solution, and a part of the droplet was exposed to air [118]. The surface tension of LM droplets can be adjusted via redox reaction. When in the system composed of LM droplet, graphite substrate, and NaOH solution, the oxidation of LM leads to the significant reduction of the surface tension of the droplet, which can induce the self-actuation and spread of LM [119]. Hu et al. [120] presented LM amoeba transformations when LM droplets were placed in such systems where the graphite can be changed to copper oxides as well. Furthermore, the LM droplet recovered to a spherical shape with the addition of Al, which can increase the surface tension via redox reaction (Figure 7E) [121].

In addition, Chen et al. [125] reported the self-growing and serpentine locomotion of LM droplets. The LM droplet was placed in the acidic copper sulfate (CuSO_4_) solution, and the redox reaction between Ga and copper ions led to the unbalanced surface tension, which further led to the localized surface pressure difference. In addition to copper ions, ferric ions can also lead to the spontaneous dispersion and large-scale deformation of LM droplets [69]. The increasing of ferric chloride concentration (FeCl_3_) and addition of acid contributes to the movement and deformation of the droplets. Furthermore, Yuan et al. [122] reported a violin-like wire oscillation behavior triggered by a LM droplet. As illustrated in Figure 7F, the copper wire was inserted inside the LM droplet containing added Al particles, then the copper wire oscillated horizontally for about half an hour. The copper wire oscillation across several LM droplets was also realized [126], and is more stable in period and direction than oscillation across a single droplet. Such results provide a unique method to fabricate self-powered oscillator machines with stable period and direction. Micro/nanodroplets can respond to chemical factors as well. Wang et al. introduced micro/nanodroplets as intelligent motors for targeted microwelding [123]. The micro/nanomotors were composed of LM cores and thin layers of platinum (Pt) half-coated on the surface of the cores (Figure 7G). The motors were self-propelled by self-electrophoresis after the addition of H_2_O_2_. In practical application, the motors tend to move along the silver nanowires and could become stuck when encountering the junctions of the network. As a result, the resistance between silver nanowires was reduced to realize the welding process. Lu et al. investigated that LM nanodroplets can fuse and degrade under a mild acidic environment [124]. The droplets were coated with doxorubicin (Dox) as transformable nanomedicine for anticancer therapy. The degradation process was as illustrated in Figure 7H; the droplets degraded within 72 h (pH 5.0). Generally, the responses to chemical factors are common and diverse. The surface tension difference induced by chemical factors leads to the large-scale transformation and locomotion of the LM droplets, which provide a theoretical basis for the realization of soft machines.

### 3.2. Electrically Induced Response

**Figure 8:**
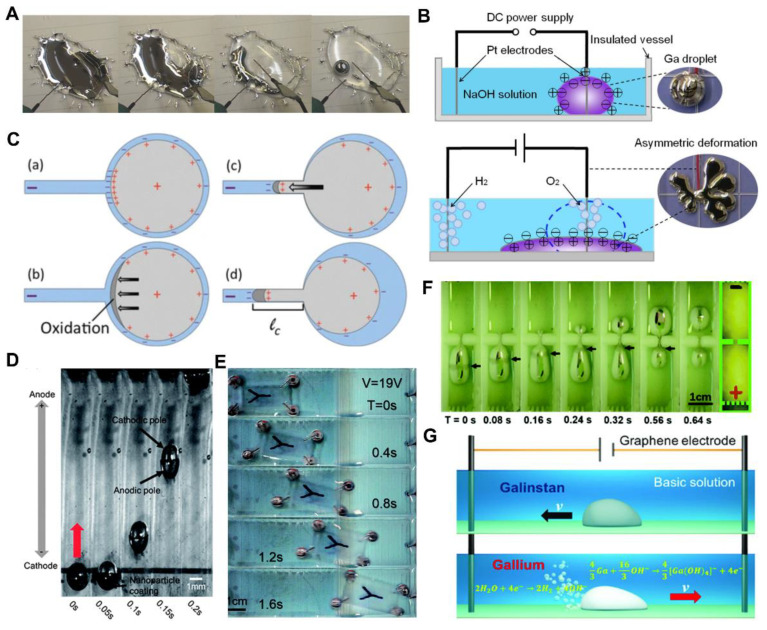
Electrically induced responses. (**A**) The electrically induced transformation of liquid metal (LM) droplet from a film into a sphere. Reprinted with permission from ref. [127]. Copyright 2014, John Wiley and son. (**B**) Schematic of the large scale deformation of the LM droplet. Reprinted from Ref. [70]. (**C**) The electrochemical actuation process of LM droplets. Reprinted from Ref. [128]. (**D**) Sequential snapshots for LM marbles moving in basic solution. Reprinted with permission from ref. [131]. Copyright 2013, The Royal Society of Chemistry. (**E**) Continuous captures of vehicle locomotion under electric field. Reprinted with permission from ref. [136]. Copyright 2016, The Royal Society of Chemistry. (**F**) Continuous captures of a LM droplet passing through a slit. Reprinted with permission from ref. [137]. Copyright 2016, The Royal Society of Chemistry. (**G**) Schematic diagram of the autonomous motion of the LM droplets with component of GaInSn and gallium, respectively. Reprinted from Ref. [138].

LM droplets can be transformed in large scale or driven under electric field. Sheng et al. [127] disclosed that the LM droplet could transform from a film into a sphere, self-rotate under control, and locomote under an electric field after being immersed in water. As illustrated in Figure 8A, when the anode and cathode were placed in the water and the LM film, respectively, the LM film transformed into a sphere after several seconds. Furthermore, several separated LM droplets located a short distance from each other could merge into a large one under the electric field. In addition, controllable self-rotation and planar locomotion under electric field were presented. Zhang et al. [70] established the electrochemical strategy, which was named SCHEME, to realize large scale reversible deformation of LM droplets. As illustrated in Figure 8B, the LM droplet was placed in NaOH solution and the anode was inserted into the droplet, while the cathode was in the solution. Under the electric field, the LM droplet spread across the bottom surface rapidly and recovered to a sphere after removing the electric field. Gough et al. [128] reported the electrochemical actuation process of LM droplets via electrocapillary actuation. As illustrated in Figure 8C, the charge distribution of the LM droplet was uneven due to the external bias voltage. The side with more positive charge was easily oxidized to form an oxide layer, which had a much lower surface tension than LM. Driven by the surface tension gradient, the LM droplet could grow into the capillary. The mechanism of above large-scale deformation and locomotion is the maragoni effect induced by the surface tension gradient. 

Furthermore, the generation of surface tension gradient can be caused by the asymmetric oxidation or electrowetting under an electric field [109,129,130]. With this basic principle, more interesting and valuable researches were carried out. Tang et al. [131] presented the electrochemically induced actuation of LM marbles. The nanoparticles coated on the surface of LM marbles formed an uneven distribution under an electric field, which would lead to novel interface characteristics (Figure 8D). This actuation behavior is affected by the type of the nanoparticles coated on the marbles’ surface. For example, the movement direction is related to the semiconductor type (p or n) of nanoparticles [132]. To further realize the antigravity motion of LM droplets under the electric field, Hu et al. [133] revealed the worm-like anti-gravity upslope LM droplet locomotion under an electric field. Because of the decreasing of surface tension due to oxidation, a LM droplet became a flat puddle when it was placed on a graphite substrate. Under an electric field, the LM droplet could creep upslope like a worm on a 10° graphite slope. By replacing the substrate with porous copper, LM droplets can be driven by the wetting difference of LM on Cu and CuGa_2_@Cu, which is completely different from the above methods [134]. By utilizing the controllable movement of LM droplets under electric field, Tan et al. [135] presented a chip cooling device in which the LM drove water to circulate with it, and then took away a lot of heat. 

More practically, LM droplets driven by an electric field can realize the function of cargo transportation and passing through the slit. Yao et al. [136] introduced a LM wheeled small vehicle which realized the controllable movement. The vehicle was composed of LM droplet wheels immersed in aqueous solutions and a plastic body, and successfully realized forward movement and steering under an electric field (Figure 8E). In addition, a LM droplet can narrow to pass through a small slit and return to its original shape under an electric field, just like a worm (Figure 8F) [137]. Furthermore, Wang et al. introduced the LM droplet with a component of Ga that could speed up in confining channels [138]. It is interesting that the movement direction of the Ga droplet under electric field is opposite to that of the Galinstan droplet due to the propulsion of the generated hydrogen bubbles (Figure 8G). As the channel width decreases, the motion velocity increases due to the enlarged electro-osmotic effect. Generally, the controllable motion of LM droplets under an electric field is interesting and meaningful, but it is still limited to be carried out in a solution environment. However, the characteristics of large-scale transformation and controllable motion still give LM droplets a good application prospect in soft machines.

### 3.3. Magnetic Field Induced Response

Magnetic fields may be used to drive both magnetic and non-magnetic materials. Liu’s group demonstrated an electrochemical LM droplet motor made by GaInSn, sodium hydroxide solution, concentric graphite electrodes, and permanent magnets. Sodium hydroxide is a powerful electrolyte and provides numerous functions in the motor system, such as removing the insulation oxide from the LM surface, reducing the motion resistance, conducting electricity, and giving impetus to the LM droplets. The rotational motion of LM droplets under electromagnetic force can be used to manufacture LM droplet motors, which allow for the transport of liquids and heat dissipation [110]. In addition, this control strategy combined with heating and/or ultrasonic devices should prove to be quite valuable in the future. Similarly, Zhang’s group proposed using a magnetic field with the relative motion of LM droplets, thus inducing a Lorentz force to drive the rotation of the droplets for heat dissipation and liquid mixing [139].

**Figure 9:**
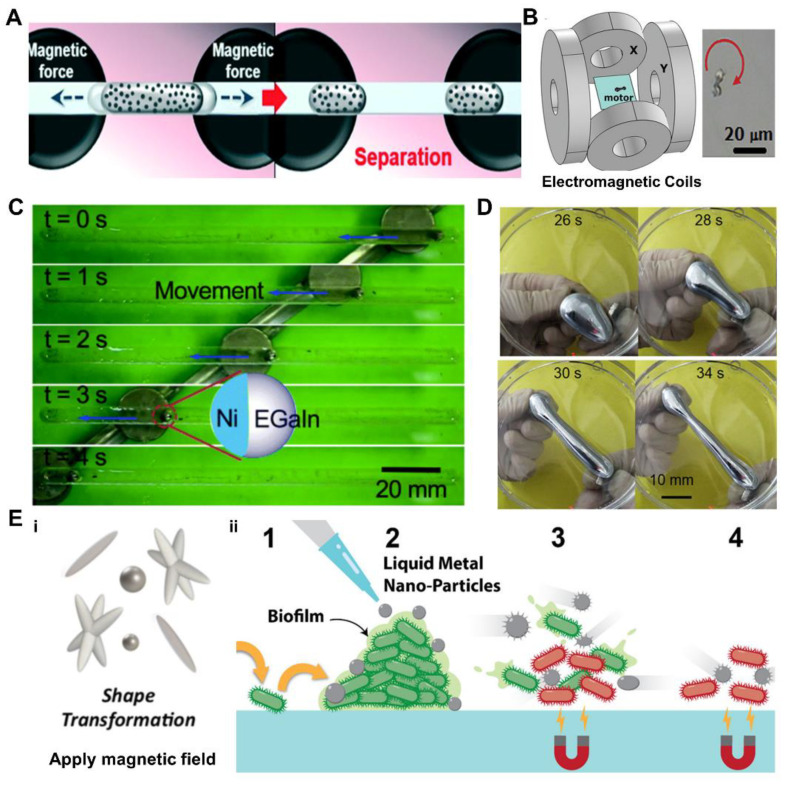
Magnetic field induced response. (**A**) Schematics depicting the separation of a liquid metal droplet in a microfluidic channel by magnetic-field-driven. Reprinted with permission from [140]. Copyright 2016, The Royal Society of Chemistry. (**B**) Schematic of magnetic liquid metal micromotors’ magnetic propulsion equipment (left) and time lapse photographs demonstrating the efficient rotation of a magnetic liquid metal micromotor (right). Reprinted with permission from ref. [141]. Copyright 2019, John Wiley and son. (**C**) The guidance of the Ni/EGaIn droplet by a magnet in a straight channel. Reprinted with permission from ref. [98]. Copyright 2016, The Royal Society of Chemistry. (**D**) The sequential snapshots of the stretching behavior of magnetic liquid metal under the magnetic manipulation in the horizontal level. Reprinted with permission from [111]. Copyright 2019, American Chemical Society. (**E**) (i). Shape transformation of magnetic liquid metal induced by magnetic field. (ii). Schematic representation of the physical action of the liquid metal-Fe particles causing bacterial cell death and reduction in the biofilm volume. Reprinted with permission from [142]. Copyright 2020, American Chemical Society.

Kim et al. [140] also demonstrated on-demand operation, separation, and merging of magnetic LM droplets in a microfluidic channel (Figure 9A). In addition to using magnetic fields to operate macroscale LM droplets, Liu et al. [141] also prepared micro- and nano- scale magnetically metamorphable micromotors (Figure 9B), which are driven by elliptically polarized magnetic fields and manufactured utilizing UV glue and ice-template transfer stencil methods. The speed and direction of the micromotor may be controlled by tuning the parameters of the magnetic field, and an alternating magnetic field can be employed in the aqueous environment for dramatic morphological alteration. It gives rise to the possibility of developing a LM droplet magnetic micromotor. LM has no natural magnetic characteristics, and Liu’s group has endowed the LM droplets with the ability to respond to magnetic fields by electroplating nickel on the surface of the LM, which can drive the movement of the LM droplets via aluminum [98], as illustrated in Figure 9C. LM droplets may be employed as novel drug transport tools for in vivo drug delivery or soft machines after being surface coated with alginate hydrogels and aluminum nanoparticles (Al-NPs).

Liu’s group also manufactured magnetic LM droplets utilizing magnetic particles and electrolytes to accomplish rapid, reversible, and large-scale movement operations in several dimensions under the magnetic field (Figure 9D), while also researching the effect of surface oxides on material deformability and mechanical strength [111]. This material was used to build electronic switches in both horizontal and vertical directions, advancing the development of LM droplet multi-degree-of-freedom actuation in free space, and laying the groundwork for subsequent fabrication of dynamically reconfigurable flexible robots. Apart from the template method, Vi Khanh Truong’s direct sonication of LM with magnetic nanoparticles results in the direct formation of particles ranging in size from 200 nm to 2 μm, and exhibiting three distinct morphologies: spheroids, rods, and stars [142]. As illustrated in Figure 9E, the particles can travel and be utilized to break the cell membranes of Pseudomonas aeruginosa and Staphylococcus aureus when triggered by alternating magnetic fields. It provides a novel solution to the problems of antibiotic resistance and antibiotic targeted therapy.

In short, magnetic field manipulation is a strategy that has numerous advantages, including maturity, safety, real-time, and non-contact operation. Due to the characteristic of LM droplets being physically manipulated and transformed in the presence of a magnetic field, paired with their excellent electrical conductivity, they can be controlled freely in the planar and vertical directions to perform magnetron circuit switching. Additionally, there are excellent properties of drug-loading, transformation, and photothermal effects through LM droplets, encouraging them to perform a variety of biomedical applications such as drug delivery, antibacterial therapy, and photothermal treatment, etc.

### 3.4. Optically Induced Response

**Figure 10:**
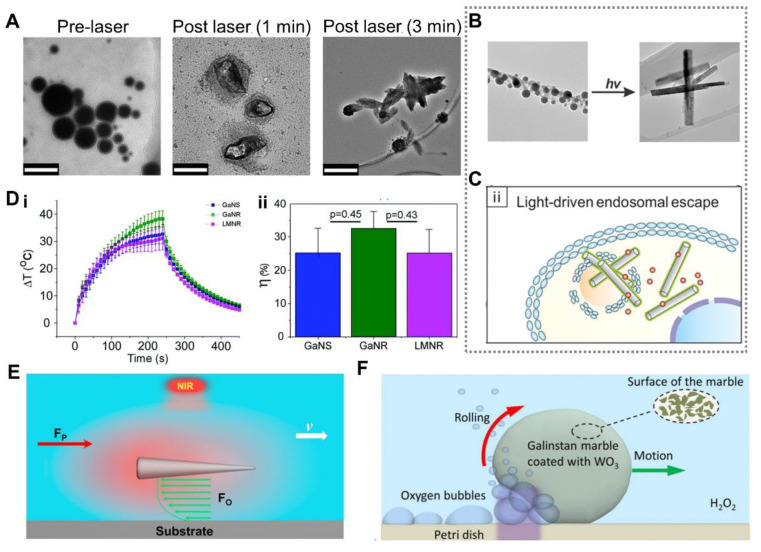
Optically Induced Response. (**A**) TEM images of liquid metal (LM) nanodroplets before and after laser irradiation. Scale bars are 200 nm, 100 nm and 500 nm, respectively. Reprinted from Ref. [145]. (**B**) TEM images of morphological transformation induced by light. Reprinted with permission from [32]. Copyright 2017, American Chemical Society. (**C**) LM nanodroplets physically disrupt endosomal membrane under light irradiation. Reprinted with permission from [32]. Copyright 2017 American Chemical Society. (**D**) (i) Average temperature elevation of Ga nanospheres, Ga nanorods, and LM nanorods irradiated under a NIR laser. (ii) Photothermal conversion efficiencies of Ga nanospheres, Ga nanorods, and LM nanorods. Reprinted with permission from ref. [147]. Copyright 2019, The Royal Society of Chemistry. (**E**) Schematic diagram of LM nanoswimmer induced by NIR light. Reprinted with permission from ref. [148]. Copyright 2021, Elsevier. (**F**) Schematic diagram of UV light induced motion of a LM marble. Reprinted with permission from ref. [72]. Copyright 2013, AIP Publishing.

LM nanodroplets with core-shell structure have been widely used as photonanomedicine for tumor photothermal therapy due to their excellent photothermal conversion [67,95]. Existing research results show that LM has good photothermal conversion efficiency under the near infrared (NIR) irradiation, especially when irradiated by an 808 nm laser [89,104,143,144]. The internal LM core usually cooperates with the functional groups modified on the surface to achieve combined treatment of tumors. In addition, LM nanodroplets were found to transform under the NIR irradiation to release anticancer drugs [145,146]. Chechetka et al. [145] found that the LM droplet size became larger under laser irradiation due to the transformation of droplets, as illustrated in Figure 10A. This provides a controlled non-contact drug release method induced by NIR irradiation. Kim et al. [112] introduced a similar transformable nanodroplet used as a vascular embolic agent and drug carrier for tumor treatment. The LM droplets could block microfluidic channels after NIR irradiation or heat treatment, and had the potential to be used as embolic agents to treat tumors that cannot be removed by surgery. Lu et al. [32] reported that endosomal escape could be enhanced by LM droplets for drug delivery. The LM droplets could transform to nanorods under light irradiation due to the transition of Ga into GaOOH, as illustrated in Figure 10B. After entering the cell via endocytosis, the LM droplets with drugs transformed to nanorods under light irradiation. The nanorods further physically disrupted the endosomal membrane to promote the endosomal escape, thereby achieving the purpose of drug release (Figure 10C). 

In addition, Sun et al. [147] reported that Ga nanorods could be used as photothermal sensitizers for tumor photothermal therapy as well. As illustrated in Figure 10D, Ga nanorods displayed more distinct temperature elevation and higher photothermal conversion efficiency than that of Ga nanospheres and LM nanorods. Furthermore, LM droplets can also be actuated by light [72,148]. Wang et al. [148] introduced a needlelike LM nano swimmer propelled by NIR light (Figure 10E). The speed of motion could reach up to 31.22 μm·s^−1^ at a laser intensity of 5 W·cm^−2^, and was related to the laser intensity and the surface potential of the substrate. Tang et al. [72] presented another way to propel the LM marbles by photochemical reaction. LM marbles coated with WO_3_ particles were placed in H_2_O_2_ solution and irradiated by ultraviolet (UV) light. The photochemical reaction triggered by UV light generated oxygen bubbles, which propelled the LM marbles (Figure 10F). 

### 3.5. Temperature Induced Response

Because of the existence of phase transition, temperature has a significant incentive effect on the behavior of LM droplets. For LM droplets in micro nano scale, the effect of temperature is more unusual. Due to the size effect, the LM droplets in the micro and nano sizes exhibit exotic physical properties. Generally, the melting points of metals decreases in line with the size of LM droplets becoming smaller. Kumar et al. [149] studied the behavior of gallium particles using differential scanning calorimetry (DSC) measurements, and the results show the melting point decreases and the super-cooling effect is more pronounced with the decrease of gallium particle size. Furthermore, Ren et al. [87] found that the melting point of GaInSn nanoparticles (≈−140 °C) is 150 °C lower than that of bulk GaInSn (≈10 °C), and the phase separation of these nanoparticles take place as the temperature decreases from room temperature to liquid nitrogen temperature. Based on the influence of size on melting point, the liquid temperature range of LM droplets can be effectively widened, which is beneficial to application.

**Figure 11:**
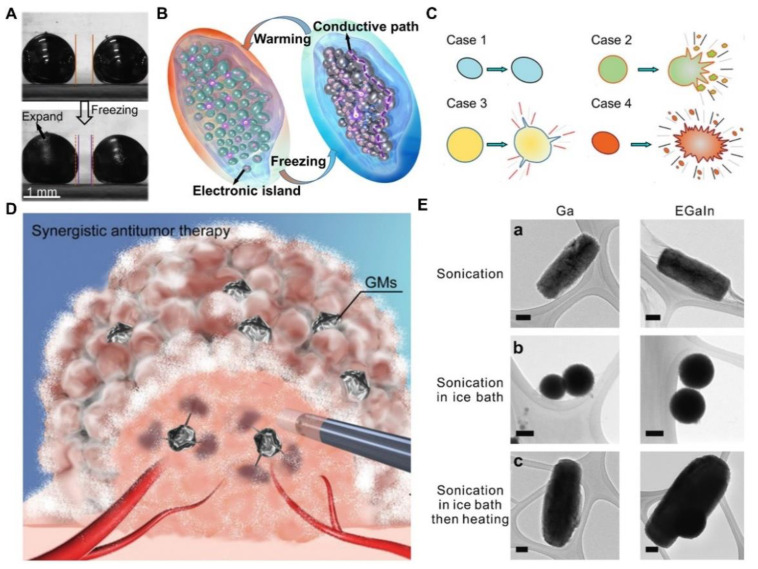
Temperature Induced Response. (**A**) Abnormal expansion of liquid metal (LM) droplets during phase transformation. Reprinted with permission from ref. [113]. Copyright, 2019, The Royal Society of Chemistry. (**B**) Schematic diagram of a functional circuit based on abnormal expansion of LM droplets. Reprinted with permission from ref. [113]. Copyright, 2019, The Royal Society of Chemistry. (**C**) Schematic illustration of four types of transformations of LM droplets induced by temperature. Reprinted with permission from ref. [71]. Copyright, 2020, American Chemical Society. (**D**) Shape change of LM droplets excited by temperature for antitumor therapy. Reprinted with permission from ref. [33]. Copyright, 2020, John Wiley and Sons. (**E**) LM droplets’ response to temperature increase. (a) Rods obtained from sonicating gallium (left column) and EGaIn (right column) in aqueous solution in the presence of Lys protein; (b) Nanospheres synthesized by repeating (a) While using an ice bath; (c) Rods obtained from post heating the spheres from (b); Here, the scale bars are 100 nm. Reprinted with permission from ref. [150]. Copyright, 2019, The Royal Society of Chemistry.

Obviously, temperature will stimulate the phase transition of LM droplets [113]. What is more unique is that the phase transition of LM droplets is accompanied by abnormal volume expansion. As illustrated in Figure 11A, the volume of LM droplets will expand when the temperature is lower than its freezing point. Such abnormal volume expansion can make LM droplets dispersed in silicone oil contact each other, thus forming a conductive path. As shown in Figure 11B, the whole process is reversible, so the material can be used for the preparation of circuits with conductive insulation transformation function. Sun et al. [71] found that low temperatures will stimulate LM droplets from ellipsoidal shape to amorphous shape (Figure 11C). This is due to the strong impulse expanded force in liquid–solid phase transition in a dual fluid system composed of LM droplets and aqueous solution, leading to a fast, large-scaled and fierce transformation. During the phase transition process, LM droplets expand to form sword-like shapes, which shows the remarkable mechanical destruction and the negligible biotoxicity, thus providing a new approach for tumor therapy. As shown in Figure 11D, a synergistic antitumor therapy of cryoablation and gallium microparticle mediated bomb-explosion-like mechanical destruction exhibits effective destructive results with a reduced recurrence rate and prolonged survival [33]. In addition, the heating process also changes the shape of LM droplets. For example, previous study indicates that spherical droplets could transform into rods after heating the solution to 70 °C for half an hour [150], as illustrated in Figure 11E. During the process of heating, gallium oxidizes to form GaOOH crystals, and the remaining alloy becomes enriched with indium due to the consumption of gallium. Considering the amazing temperature response, LM droplets with different shapes can be formed by changing the ambient temperature, which is favorable for potential applications. More in-depth study on the matter should be conducted. 

### 3.6. Ultrasound Induced Response

**Figure 12:**
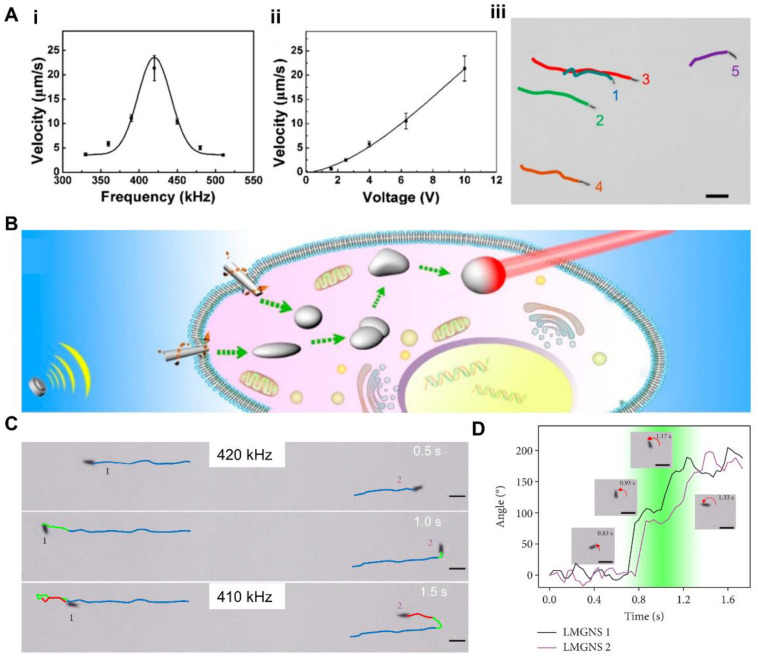
Ultrasound Field Induced Response. (**A**) Acoustically propelled liquid metal gallium nanomachine (LGNMs). (i) Average velocity of LGNMs under ultrasound of different frequencies (U = 10 V); (ii) Average velocity of LGNMs under different voltages at a frequency of 420 kHz; (iii) Time-lapse images of five LGNMs with different lengths. Reprinted with permission from [34]. Copyright 2018, American Chemical Society. (**B**) Schematic diagram of photothermal therapy achieved by acoustically propelled LGNMs. Reprinted with permission from [34]. Copyright 2018, American Chemical Society. (**C**) The time-lapse images of direction control of LMGNSs by frequency of ultrasound. Reprinted from Ref. [151]. (**D**) The motion direction changes during frequency changing. Reprinted from Ref. [151].

Currently, LM nanodroplets are investigated to be propelled by ultrasound [34,151]. Wang et al. [34] introduced a rodlike LM gallium nanomachine (LGNM) which could swim under ultrasound due to the propulsion of the acoustic radiation force. As illustrated in Figure 12A, the velocity of motion depended on the frequency and voltage of the ultrasound. In addition, the velocity was also related to the length of LGNMs. The velocity could reach up to about 23 μm·s^−1^ at a frequency of 420 kHz. Wang et al. used LGNMs as nanomedicines for the photothermal therapy of tumors. In this application, LGNMs were propelled to move autonomously by an ultrasound field, as illustrated in Figure 12B. 

Furthermore, the motion direction was determined to be controllable under ultrasound of different frequencies. Wang et al. [151] introduced a leukocyte membrane-coated gallium nano-swimmer (LMGNS), which can also be propelled by ultrasound. As illustrated in Figure 12C, two LMGNSs moved in reverse directions in 1 s when the ultrasound frequency changed from 420 kHz to 410 kHz. Figure 12D shows the specific process of changing direction over time. The maximum velocity of LMGNS could reach over 100 μm·s^−1^ under the frequency ranging from 415 to 425 kHz. The LMGNSs could actively internalize into the tumor cells for combined photothermal and chemical therapy of tumors. The motion propelled by ultrasound provides a novel strategy for the contactless propulsion of LM nanodroplets. 

## 4. Conclusions and Outlook

LM droplets with rich properties at multiple scales are attracting extensive attention. The past ten years have brought remarkable advances in the controllable preparation of LM micro/nano droplets and the development of responsive smart LM droplets. So far, various methods for preparing LM droplets have been successfully proposed. The intelligent response behaviors of LM droplets under various external fields have also been widely studied. Along this path, further research is still ongoing. To guide follow-up study, opportunities and challenges in the development of responsive LM droplets will be pointed out and summarized here.

Firstly, the preparation of LM droplets is the first step in conducting study, and it is still meaningful to explore the controllable mass preparation of smaller scale LM nano droplets. Previously obtained nanoscale LM droplets are generally in organic solvents, which in fact lose their external field response to a certain extent due to the existence of surface oxide film. However, with the removal of surface oxide film, the merging of droplets is difficult to avoid. Such challenges still exists, awaiting further research to help overcome them.

Secondly, giving specific functions to the response behavior of LM droplets is vital for potential applications in the future. Achieving higher response accuracy of LM droplets to external factors is beneficial for this goal. The driving force of external fields on LM droplets is relatively small, making it challenging for LM droplets to complete specific tasks in some cases. Furthermore, it should be stipulated that our full understanding of the LM droplet is still far from being sufficient. As revealed recently [152], the LM droplet machine rotating under electrical actuation, or the self-fueled LM motor, even run as an endogenous fluidic magnet owing to its electromagnetic homology. This suggests a new clue with which to tackle the classical physical puzzle lying behind the magnetic monopoles. Clearly, the more complex response behavior under multi field coupling is also of great benefit to the application of LM droplets.

Finally, the underlying mechanisms behind the various response behaviors of LM droplets are still unclear, and their theoretical studies are still insufficient. In fact, theory can not only explain experimental phenomena, but also contribute to precise prediction and regulation of response behavior, which is of great significance for possible practical applications. In follow-up studies, the theory behind the phenomenon should be given special attention. For example, due to the unique properties of LM, should the traditional electric double layer theory be improved to more accurately describe the Marangoni flow of LM droplets? This similar theoretical question deserves constant consideration and advancement in subsequent research.

Overall, compared with traditional droplets, LM droplets with multiple properties show rich and extraordinary response behaviors. External stimuli that have been implemented so far include, but are not limited to, electric, magnetic, acoustic, light, thermal, and chemical fields. The ultimate goal of exploring the response behavior of LM droplets is to realize their application, and follow-up efforts around this point are essential. In the future, it is believed that responsive LM droplets will usher in a new stage of development and open new doors for many important applications, such as soft robots, drug delivery, and so on.

## Figures and Tables

**Table 1:** The physical properties of common liquid metals [39,47,50].

	Ga	GaIn_24.5_ (EGaIn)	Ga_67_In_20.5_Sn_12.5_ (Galinstan)	Ga_61_In_25_Sn_13_Zn_1_
Melting point (°C)	29.8	15.7	10.5	7.6
Boiling point (°C)	2204	2000	1300	900
Density (10^3^ kg/m^3^)	6.08	6.28	6.36	6.5
Viscosity (10^−7^ m^2^/s)	3.24	2.7	2.98	0.71
Surface tension (N/m^1^)	0.72	0.624	0.533	0.5
Conductivity (10^6^ S/m)	3.7	3.4	3.1	2.8
Thermal conductivity (W/m °C)	29.4	42.2	44.8	48.2

**Table 2:** The summary of the response characteristics of responsive liquid metal droplets.

Response Factors	Mechanism	Characteristics	Applications
Chemical field	Marangoni effects [108],	Self energy supply,	Soft robots,
	Interfacial tension gradient	large scale deformation,	drug delivery,
Electric field	Solution viscous force drive [109]	Good controllability	Cargo transportation
Magnetic field	Lorentz force [110],	Non-contact manipulation,	Reconfigurable electronics,
	Magnetic force [111]	large driving force	antibacterial
Light	Heat generation [112],	Photothermal synergy,	Photothermal therapy,
	bubble propulsion [72]	non-contact	drug release
Temperature field	Phase transition [113]	Rapid response	Functional circuit, tumor therapy
Acoustic field	Acoustic radiation force [34]	Biocompatible	Photothermal cancer cell therapy

## Data Availability

No new data were created or analyzed in this review.
